# Oral health and HIV infection among female sex workers in Abidjan, Côte d’Ivoire

**DOI:** 10.1186/s12903-015-0129-0

**Published:** 2015-12-02

**Authors:** Marcellin N. Nouaman, David G. Meless, Patrick A. Coffie, Elise Arrivé, Boris K. Tchounga, Didier K. Ekouévi, Camille Anoma, Serge P. Eholié, François Dabis, Antoine Jaquet, the IeDEA West Africa collaboration

**Affiliations:** Programme PACCI, CHU Treichville, Site de Recherche ANRS, Abidjan, Côte d’Ivoire; UFR Odonto-Stomatologie, Université Félix Houphouët Boigny, 18 BP 1954 Abidjan 18, Côte d’Ivoire; Département de Dermatologie et Infectiologie, UFR des Sciences Médicales, Université Félix Houphouët Boigny, Abidjan, Côte d’Ivoire; Université Bordeaux, ISPED, Centre INSERM U897- Epidémiologie-Biostatistique, F-33000 Bordeaux, France; INSERM, ISPED, Centre INSERM U897- Epidémiologie-Biostatistique, F-33000 Bordeaux, France; Département de santé publique, Faculté des Sciences de la santé, Université de Lomé, Lomé, Togo; Clinique de Confiance, Abidjan, Côte d’Ivoire

**Keywords:** HIV/AIDS, Oral-mucosal lesions, Dental caries, Periodontitis, Female sex workers, Alcohol use, Africa

## Abstract

**Background:**

Worldwide, female sex workers (FSW) represent a vulnerable population for oral diseases due to many risk factors including HIV infection and drug abuse. In sub-Saharan Africa, little is known about the burden of oral diseases and their determinants in vulnerable populations. The aim of the study was to estimate the prevalence and associated factors of oral diseases among FSW.

**Methods:**

A cross sectional study was conducted among FSW who attended a dedicated non-profit clinic in Abidjan, Côte d’Ivoire from June to August 2013. Data about the presence of dental caries, periodontitis and oral-mucosal lesions were collected by a dentist during an oral examination. Behavioural information related to oral hygiene habits as well as tobacco and alcohol consumption were collected through a standardized questionnaire. Information related to HIV infection including HIV diagnosis, last known CD4 count and antiretroviral therapy were documented through a medical chart review. Logistic regression models were used to identify factors associated with oral diseases.

**Results:**

A total of 249 FSW with a median age of 29 years, [Inter Quartile Range (IQR) = 23–36] and a median duration of sex work of 24 months [IQR 9–60]) were included. Current tobacco use and hazardous alcohol use were reported in 21.7 % and 19.7 % of FSW, respectively. The estimated prevalence of HIV infection was 33.7 % [95 % confidence interval (CI); 27.8 – 39.6]) and 82.1 % of HIV-infected FSW were on antiretroviral therapy . The prevalence of dental caries, periodontitis and oral-mucosal lesions were 62.3 % [95 % CI 55.5 – 67.5], 14.5 % [95 % CI 10.2 – 18.9] and 8.2 % [95 % CI 4.8 – 11.5], respectively. In multivariate analysis, periodontitis, oral-mucosal lesions and HIV infection were associated with odds ratio of 2.6 [95 % CI, 1.2–5.8]) and 50.0 [95 % CI; 6.4–384.6].

**Conclusions:**

This study showed a high prevalence of oral diseases among FSW in Abidjan. HIV infection was common and significantly associated with periodontal diseases and oral-mucosal lesions. There is a need to integrate regular screening and treatment of oral lesions into the medical follow-up of FSW along with strategies for HIV prevention.

**Electronic supplementary material:**

The online version of this article (doi:10.1186/s12903-015-0129-0) contains supplementary material, which is available to authorized users.

## Background

Sub-Saharan Africa remains one of the hardest hit regions of the world for HIV infection with about 4.9 % of adults 15 – 49 years living with HIV in 2013 [1]. Côte d’Ivoire, harbours one of the highest numbers of HIV-infected persons in West Africa with an average HIV prevalence estimated at 3.2 % [2.8–3.8 %] in 2013 [1]. Vulnerable populations such as female sex workers (FSW) are more likely to be infected with HIV. In sub-Saharan Africa, many countries report an estimated HIV prevalence ≥ 25 % among FSW [2, 3].

Oral diseases are defined as any morphological or functional abnormalities teeth and supporting structures. These are mainly dental caries resulting from the progressive decalcification of tooth hard tissue, periodontal disease due to inflammation of tooth-supporting tissues and pathologies of the oral mucosa. Oral diseases affect the majority of adult populations worldwide but are particularly high and burdensome in socially marginalized populations due to their limited access to prevention and care [4]. Several studies in sub-Saharan Africa reported a high prevalence (of the order 40.8 to 86.2 %) of oral diseases among school children and adults [5, 6]. In Côte d’Ivoire, epidemiologic reports on oral health are scarce, but a high prevalence of oral diseases is expected in vulnerable populations [7]. In addition to common etiological factors such as poor oral hygiene, consumption of refined carbohydrates and limited access to dental care [8], HIV infection is a potential determinant of poor oral health. Indeed, several oral-mucosal lesions of viral, bacterial or fungal origin are known to be associated with HIV infection, some of which contribute to the HIV/AIDS clinical staging according to the US Centers for Disease Control (CDC) classification [9–11]. Excessive alcohol consumption, tobacco use and others addictives behaviours associated with a poor oral health are also more frequent among vulnerable populations such as FSW and people living with HIV [12–14]. Therefore, FSW constitute a high-risk population for oral diseases many of which being preventable, treatable or curable. The objectives of this study were to estimate the prevalence of oral diseases including dental caries, periodontal disease and HIV-associated oral-mucosal lesions and to identify their associated factors among FSW in Abidjan.

## Methods

### Study design

A cross-sectional study was conducted among consenting adult FSW over a three month period, from June to August 2013.

### Setting

This study was conducted at the “Clinique de Confiance” in Abidjan, Côte d’Ivoire. The “Clinique de Confiance” is a nongovernmental organization (NGO) dedicated to the overall prevention and care of HIV infection and other sexually transmitted infections among FSW and men having sex with other men (MSM) in the urban area of Abidjan. To date, this NGO ensures the follow-up of more than 1 500 FSW and MSM.

### Study population

All adult FSW (≥18 years) who attended this clinic during the study period and who provided their signed inform consent were eligible to participate. About 20 FSW come to the clinic in daily consultation. Since it was not possible to include all the FSW, a number from 1–20 were sequentially assigned to women. Thus, all FSW having an odd number were included in the study in case of acceptance. In case of refusal, the following odd number was selected.

### Variables

#### Oral-health

Dental status was assessed using the Decayed, Missing and Filled Teeth (DMFT) index as described by Klein and Palmer [15]. The World Health Organization (WHO) defines a standard DMFT scoring scale that qualifies the needs for dental care: very low (0.1 ≤ DMFT index <1.2); low (1.2 ≤ DMFT index ≤ 2.6); moderate (2.7 ≤ DMFT index ≤4.4); high (4.5 ≤ DMFT index ≤6.5) and very high (DMFT index > 6.5). A D index (number of carious teeth) ≥1 indicates at least one carious lesion.

The periodontal status was assessed based on the “Community Periodontal Index” (CPI). The dentition was divided into six sextants: 17 to 14, 13 to 23, 24 to 27, 47 to 44, 43 to 33, 34 to 37. We assigned a numeric code to each sextant based on the examination of 10 indicator teeth (17/16, 1 l, 26/27, 47/46, 31, 36/37). A sextant was considered only if it had at least two functional teeth. The highest code per sextant was reported [16].

Subjects were then classified into five categories defined by the codes: Code 0 = healthy gum; Code 1 = presence of bleeding; Code 2 = presence of calculus; Code 3 = presence of pockets of 4 to 5 mm; Code 4 = pocket over 6 mm. Presence of pockets was identified by periodontal probing in six sites per tooth (mesio-lingual, centro-lingual, disto-lingual, mesio-buccal, centro-buccal and disto-buccal) [16]. A CPI score ≥3 was retained to define the presence of periodontitis.

HIV-related oral-mucosal lesions were screened including oral candidiasis, oral hairy leukoplakia and Kaposi’s sarcoma according to Oral HIV/AIDS Research Alliance case definitions [17].

Oral hygiene level was assessed using the Oral Hygiene Index Simplified (OHIS) [18]. The OHIS is the sum of the debris index and the calculus index. Both debris index and calculus index are the means of six debris (calculus) scores assessed on six tooth surfaces: 0 = No debris (calculus); 1 = Debris (calculus) on less than 1/3 of the tooth surface; 2 = Debris (calculus) covering between 1/3 and 2/3; 3 = Debris (calculus) covering more than 2/3. The OHIS is assessed by taking six reference teeth (11, 16, 26 of maxillary and 31, 36, 46 in the mandible). The OHIS was categorised into a quantitative ordinal variable with four modalities. Oral hygiene was defined as follows: excellent if OHIS = 0; good if (0.1 < OHIS <0.9); fair if (1 ≤ OHIS ≤1.9) and poor if (OHIS ≥2). In our study, oral hygiene was defined as a three-modality variable by merging the “excellent” and “good” categories.

### Socio-demographic and behavioural characteristics

Socio-demographic characteristics including date of birth and education level as well as addictive behaviours (tobacco, alcohol and other drugs consumption) were collected. Tobacco consumption was reported in three modalities “never smokers”, “present or past smokers < 10 cigarettes/day” and “present or past smokers ≥ 10 cigarettes/day”. The level of alcohol use was scored using the Alcohol Use Disorders Identification Test (AUDIT) developed by the World Health Organisation (WHO) [19]. This 10-item questionnaire assessed alcohol use in the past 12 months based on a 40 points scale. Participants were classified into three modalities: no alcohol user, moderate alcohol user (AUDIT score < 8) or hazardous alcohol users (AUDIT score ≥ 8). Characteristics related to the professional activity of FSW were also collected. These included the number of working days per/week as sex workers, the number of customers per/day in the last working week, use of condom, the existence of a regular partner, the type of sexual practices and the duration of sex work.

The daily number and the average duration of toothbrushing were also collected. The quality of brushing was defined as “low” if the FSW reported brushing her teeth once a day and never in the evening after dinner; “acceptable” if the FSW reported brushing her teeth once a day, in the evening after dinner or at least twice a day before or after meals; “good” if the FSW reported brushing her teeth twice or thrice daily after eating.

### HIV infection

The “Clinique de Confiance” systematically offers HIV testing to all FSW attending the clinic, in line with recommendations of the national AIDS program. Dates and results of the last HIV test were registered for all participating FSW. For HIV-infected FSW, additional information was collected including the use of antiretroviral therapy (ART), date of ART initiation, current ART regimen, last known CD4 cell count and CDC clinical stage at first entry into care for their HIV infection.

### Data collection

Oral health-related variables were derived from data collected on a standardized form during an oral clinical examination performed by a unique dental surgeon previously trained to the screening of oral diseases, especially oral-mucosal lesions. Oral examination was performed using an overhead dental light. Dental caries were assessed with a probe number 6. The CPI was assessed using a dedicated non-invasive probe with a ball-shaped end of 0.5 mm diameter and a coloured portion of 3.5 mm to 5.5 mm. The dental surgeon was trained for the screening of oral-mucosal lesions using a module designed and developed by the Oral HIV/AIDS Research Alliance [17]

Variables related to socio-demographic characteristics and addictive behaviours were collected through face-to-face interview conducted by a dental surgeon using a standardised questionnaire before the oral examination.

HIV-related variables were collected by reviewing clinical medical records of HIV-infected FSW participating to the study.

### Data analysis

Qualitative variables were described in terms of number and percentage and quantitative variables in terms of mean [standard deviation (SD)] and median [interquartile range (IQR)]. Comparisons were performed using Student’s *t* test, nonparametric Mann–Whitney U test, or analysis of variance for continuous variables. The Pearson’s Chi-square test or the Fisher’s exact test was used to compare frequencies when appropriate.

Three unconditional logistic models were used to identify factors associated with the presence of dental caries, periodontitis and oral-mucosal lesions. Variables statistically associated with these oral lesions with a *p*-value <0.20 in univariate analysis were retained and introduced in the initial multivariate model. A backward stepwise regression procedure was then applied to select the final multivariate models. Adjusted Odds Ratio (aOR) were estimated with their 95 % confidence intervals (CI). A *p*-value < 0.05 was considered for statistical significance.

All analyses were performed with Stata software (StataTM 9.0 College Station, Texas, USA).

### Ethical aspects

Before being included, all FSW were informed about risks and benefits associated to their participation in the present study. All FSW provided their written consent to participate. Data collected through this study were part of the IeDEA study Protocol in Côte d’Ivoire (http://mereva.isped.u-bordeaux2.fr/iedea/) already approved by the National Ethics Committee of Côte d’Ivoire. Oral examination was part of the routine clinical care delivered at the “Clinique de Confiance” during the study period. Regardless to their study participation, all FSW attending the clinic were able to access to this oral examination, if needed (Additional file 1).

## Results

### Socio-demographic and behavioural characteristics

During the study period, 523 FSW attended the clinic. Among them, 258 (49, 3 %) were randomly selected to participate in the study. A total of nine FSW were excluded: one refused to participate to the study and eight refused to be tested for HIV infection. Their median age of the 249 FSW included in our analysis was 29 years [IQR = 23–36], 54 (21.7 %) were current smokers and 16 (6.4 %) former smokers (Table 1). In those reporting a present or past history of tobacco smoking, the median number of cigarettes smoked per day was 10 [IQR = 5–20]. Alcohol use in the past 12 months was reported by 133 (53.4 %) and six (2.4 %) reported smoking cannabis.

**Table 1:** Socio-demographic, behavioural characteristics according to HIV status among 249 female sex workers followed up at the “Clinique de Confiance”, Abidjan, 2013

Variable	HIV+ *n* = 84	HIV- *n* = 165	Total *n* = 249	**p**
			*n* (%)	
Socio-demographic characteristics				
Median Age (IQR) (years)	36.5[32 42]	25[23 30]	29[23 36]	
Education level				**<10** ^**−2**^
None	32(38.1)	57(34.6)	89(35.7)	
Primary education	38(45.2)	46(27.8)	84(33.8)	
Secondary and further education	14(16.7)	62(37.6)	76(30.5)	
Sexual behaviour				
Duration of sex work				**<10** ^**−2**^
<2 years	14(16.7)	87(52.7)	101(40.6)	
≥2 years	70(83.3)	78(47.2)	148(59.4)	
Number of working days/week^$^				**<10** ^**−2**^
1 – 4 days	27(32.1)	26(15.8)	53 (02.8)	
≥5 days	57(67.9)	139(84.2)	196(78.7)	
Number of customers/day^$^				**0.02**
1 – 4 customers	25(29.8)	29(17.6)	54(21.7)	
≥5	59(70.2)	136(82.4)	195(78.3)	
Condom use^$^				**<10** ^**−2**^
Never	5(6.0)	3(1.9)	8(3.2)	
Sometimes	29(34.5)	23(13.9)	52(20.9)	
Always	50(59.5)	139 (84.2)	189(75.9)	
Usual type of sexual activity with customers				**0.73**
Vaginal sex exclusively	72(85.7)	144(87.3)	216(86.7)	
Vaginal, oral and/or anal sex	12(14.3)	21(12.7)	33(13.3)	
Addictive products				
Tobacco consumption				**0.69**
Never smokers	63(75.0)	116(70.3)	179(71.9)	
Present/past smokers <10 cig/day	8(9.5)	21(12,7)	29(11,7)	
Present/past smokers ≥10 cig/day	13(15.5)	28(17,0)	41(16.4)	
Alcohol consumption				**0.17**
No	43(51.2)	73(44.2)	116(46.6)	
Yes audit score < 8	30(35.7)	54(32.8)	84(33.7)	
Yes audit score ≥ 8	11(13.1)	38(23.0)	49(19.7)	
Oral hygiene				
Past history of dental consultation				**<10** ^**−2**^
Yes	64(76.2)	155(93.9)	219(87.9)	
Quality of tooth brushing				**0.02**
Insufficient	66(78.6)	148(89.7)	214(85.9)	
Good	18(21.4)	17(10.3)	35(14.1)	
Oral hygiene index				**0.01**
[0 0.9]	14(17.0)	55(33.3)	69(27.9)	
[1 1.9]	29(35.4)	58(35.2)	87(35.2)	
≥2	39(47.6)	52(31.5)	91(36.8)	

**p**: ***p*** **-value**, a *p*-value < 0.05 was considered for statistical significance

$: In the past week

*IQR* interquartile range

The systematic use of condom was reported by 189 (75.9 %) of the participating FSW.

### Characteristics of HIV infection

The estimated prevalence of HIV infection was 33.7 % [95 % CI 27.8 – 39.6]. Of the 84 HIV-positive FSW, 97.6 % were infected with HIV-1, 19.1 % were at stage C (CDC clinical stage) and 82.1 % were on ART with a median time on ART of 4 years [IQR = 1–5]. The two most common ART regimens were zidovudine/lamivudine/nevirapine (65.2 %) and tenofovir/lamivudine (or emtricitabine)/efavirenz (21.7 %) (Table 2).

**Table 2:** Main characteristics of HIV infection among 84 HIV-positive female sex workers followed up at the “Clinique de Confiance”, Abidjan, 2013

	*N* = 84	(%)
Type of HIV		
HIV-1	82	(97.6)
HIV-1 + 2 dually infected patients	2	(2.4)
Time since first positive HIV serology, Median [IQR]^≠^ (years)	4.1	[1 5.7]
ART^β^ use,		
Yes	69	(82.1)
No	15	(17.9)
ART regimen^a^		
Zidovudine/Lamivudine/Nevirapine	45	(65.2)
Zidovudine/Lamivudine/Efavirenz	4	(5.8)
Zidovudine/Lamivudine/Lopinavir/Ritonavir	1	(1.5)
Tenofovir disoproxil fumarate/Emtricitabine/Efavirenz	9	(13.0)
Tenofovir disoproxil fumarate/Lamivudine/Efavirenz	6	(8.7)
Tenofovir disoproxil fumarate/Emtricitabine/Nevirapine	4	(5.8)
CD4 count^†^ - Median [IQR] in cells/mm^3^	366	[260 545]
On ART (*n* = 69)	338	[97 459]
No ART (*n* = 15)	649	[504 825]
CDC Clinical stage^∫^		
Stage A	29	(34.5)
Stage B	39	(46.4)
Stage C (AIDS)	16	(19.1)

^**≠**^ IQR: Interquartile Range; ^β^ART = Anti-Retroviral Therapy; ^a^ART regimen at study enrolment (*N* = 69); ^†^Last known CD4 count in cells/mm^3^; ^∫^HIV/AIDS clinical stage at first entry into care according to the US Centers for Disease Control and Prevention classification

### Oral diseases

The mean OHIS was 1.6 (SD 0.1) and 91 (36.8 %) FSW were considered to have a “poor” OHIS. The estimated prevalence of dental caries was 62.3 % [95 % CI; 55.5 – 67.5] with a DMFT index of 3.5 (SD 4.4) (Table 3). The presence of at least one dental carie was reported in 60 (71.4 %) HIV-infected FSW and 95 (57.6 %) HIV-uninfected FSW, (*p* =0.03). In multivariate analysis, factors associated with dental caries were a past history of dental consultation (aOR = 6.4; [95 % CI; 1.8–22.3]) and an OHIS between 1 and 1.9 (aOR = 2.5; [95 % CI; 1.3–5.0]). No significant association was found between tobacco, alcohol use, HIV infection and dental caries; and between dental caries and HIV infection (Table 4).

**Table 3:** Dental index and oral diseases according to HIV and ART status among female sex workers followed up at “Clinique de Confiance”, Abidjan, 2013

		HIV+ (*n* = 84)	HIV- (*n* = 165)	Total *n* = 249	*p*	ART (*n* = 69)	No ART (*n* = 15)	Total (*n* = 84) &	*p*
				n (%)					
Dental Index									
DMFT index^$^ M(SD)	3.5 (4.4)	5.6 (5.6)	2.5(3.1)		<10^−2^	5.7 (5.7)	5.0 (5.3)		0.6
Decayed teeth M(SD)	2.3 (2.9)	3.3 (3.5)	1.9 (2.4)		<10^−2 ^	3.3 (3.5)	3.1 (3.4)		0.8
Missing teeth M(SD)	1.1 (2.8)	2.3 (4.2)	0.6 (1.3)		<10^−2^	2.4 (4.4)	1.9 (3.2)		0.7
CPI index*					0.02				0.4
Score: 0		6(7.3)	15(9.1)	21 (8.5)		6(8.9)	0(0)	6(7.32)	
Score: 1		18(22.0)	59(35.8)	77 (31.2)		15(22.4)	3(20.0)	18(21.95)	
Score: 2		38(46.3)	75(45.4)	113 (45.8)		28(41.9)	10(66.6)	38 (46.34)	
Score: 3		15(18.3)	13(7.9)	28 (11.3)		14(20.9)	1(6.7)	15(18.29)	
Score: 4		5(6.1)	3(1.8)	8 (3.2)		4(5.9)	1(6.7)	5(6.10)	
Oral diseases									
Carious lesions					0.03				0.6
Yes		60(71.4)	95(57.6)	155(62.3)		50(72.5)	10(66.7)	60(71.4)	
Periodontitis^¥^					<10^−2^				0.2
Yes		20(24.4)	16(9.7)	36(14.6)		18(26.9)	2(13.3)	20(24.4)	
Oral-mucosal lesions					<10^−2^				0.08
Yes		20(23.8)	1(0.6)	21(8.4)		19 (27.5)	1(6.7)	20(23.8)	

*p*: < 0.05 was considered for statistical significance

0: healthy gum

1: presence of bleeding

2: presence of calculus

3: presence of pockets of 4 to 5 mm

4: pocket over 6 mm

*: CPI : Community Periodontal Index

^**$**^Decayed, Missing and Filled Teeth index ; M(SD) = mean (standard deviation)

&: Two missing data during analysis

¥: Periodontitis = CPI index ≥ 3 (CPI = Community Periodontal Index)

**Table 4:** Factors associated with the presence of oral diseases among female sex workers followed up from June to August 2013 at “Clinique de Confiance”, Abidjan, 2013

Variable	n/N	Periodontitis (*N* = 247)^a^			n/N	Carious lesions (*N* = 247)^a^			n/N	Oral-mucosal lesions (*N* = 247)^a^		
		*Multivariate analysis*				*Multivariate analysis*				*Multivariate analysis*		
		aOR	95 % CI	*P*		aOR	95 % CI	*P*		aOR	95 % CI	*P*
Past history of dental consultation		-	-	-				<10^−2^		-	-	-
No					129/219	1						
Yes					25/28	6.4	[1.8 22.3]					
Tobacco consumption				0.30				0.28				0.39
Non-smokers	27/178	1.0			112/178	1.0			17/178	1.0		
Smokers < 10 cig/d	2/28	0.30	[0.1 1.4]		18/28	0.8	[0.3 2.0]		1/28	0.5	[0.1 5.1]	
Smokers ≥ 10 cig/d	7/41	0.60	[0.2 1.7]		24/41	0.5	[0.2 1.2]		2/41	0.6	[0.1 3.4]	
Alcohol consumption				0.12				0.60				0.30
No	15/115	1.0			78/115	1.0			12/115	1.0		
Yes audit score <8	11/83	1.2	[0.4 2.9]		41/83	0.5	[0.3 0.9]		7/83	0.8	[0.3 2.3]	
Yes audit score ≥8	10/49	2.9	[0.9 9.0]		35/49	1.1	[0.5 2.5]		1/49	0.3	[0.0 3.0]	
HIV serologic status				0.01		-	-	-				<10^−2^
HIV-	16/165	1.0							1/165	1.0		
HIV+	20/82	2.6	[1.2 5.8]						19/82	50.0	[6.4 384.6]	
Oral hygiene index				<10^−2^				0.04				0.06
[0 0.9]	2/69	1.0			35/69	1.0			2/69	1.0		
[1 1.9]	10/87	3.4	[0.6 16.4]		60/87	2.5	[1.3 5.0]		5/87	1.4	[0.2 8.7]	
≥2	24/91	9.8	[2.1 44.2]		59/91	2.0	[1.0 3.8]		13/91	3.8	[0.7 19.6]	

^a^Two missing data during multivariate analysis

*aOR* adjusted odds ratio, *CI* confidence interval, *P* adjusted *p*-value

Overall, 235 (91.4 %) FSW had periodontal disease. The prevalence of periodontitis was 14.5 % [95 % CI; 10.2 – 18.9]. HIV-infected FSW were more likely to presented with periodontitis than HIV-uninfected FSW with figures of 20(24.4 %) and 16 (9.7 %) respectively, (*p* <10^−2^) (Table 1). In multivariate analysis, the presence of periodontitis was associated with HIV infection (OR = 2.6; [95 % CI; 1.2–5.8]) and an OHIS ≥ 2 (OR = 9.8; [95 % CI; 2.1–44.2]). No association was found between tobacco or alcohol use and the presence of periodontitis.

Oral-mucosal lesions were diagnosed in 21 (8.4 %) FSW. Oral candidiasis was identified in 14 (66.6 %) cases including five pseudomembranous candidiasis cases and nine erythematous candidiasis cases. The remaining clinical diagnoses of oral-mucosal lesions were reported as follows: Herpes simplex (*n* = 2), recurrent aphthous stomatitis (*n* = 2), oral hairy leukoplakia (*n* = 1), oral warts (*n* = 1) and primary herpetic gingiva-stomatitis (*n* = 1). An oral-mucosal lesion was diagnosed in 20 (23.8 %) HIV-infected FSW and one (0.6 %) HIV-uninfected FSW (*p* <10^−2^). The median CD4 count was 273 [IQR = 222–404] cells/mm^3^ in the 20 HIV- infected FSW with oral-mucosal lesions versus 409 [IQR = 290–581] cells/ mm^3^ in the 64 HIV-infected FSW without any oral-mucosal lesions (*p* = 0.01). Among HIV-infected FSW on ART, the prevalence of oral-mucosal lesions was 28.0 % in those on AZT-containing ART regimen versus 26.3 % in those on TDF-containing ART regimen (*p* = 0.8). In multivariate analysis, HIV infection was significantly associated with the presence of oral-mucosal lesions (OR = 50.0; [95 % CI; 6.4–384.6]).

## Discussion

This study conducted among FSW followed in the urban area of Abidjan, Côte d’Ivoire showed a high prevalence of oral diseases. The prevalence of HIV infection was high and significantly associated with the presence of periodontitis and oral-mucosal lesions. The prevalence of HIV infection was concordant with a previous reports from Côte d’Ivoire where the prevalence of HIV among FSW ranged from 26.6 to 33.0 % [3, 20]. The high prevalence of oral-mucosal lesions among HIV-infected FSW is largely related to the presence of infections known to be associated with HIV such as oral candidiasis. Despite the lack of precision reflected by the wide confidence interval, the elevated odd ratio showed the particularly high association between the presence of oral-mucosal lesions and HIV infection. Immunodeficiency is a known risk factor for the occurrence of oral-mucosal lesions [21]. Indeed, HIV-infected FSW with oral-mucosal lesions had a significantly lower number of CD4 cell count compared to HIV-infected FSW without oral-mucosal lesions. Therefore, the prevalence of oral-mucosal lesions in our population of FSW accessing to ART was lower compared to previous reports conducted among HIV-infected persons with limited or no access to ART. In South Africa, Bhayat et al. reported that 53 % of HIV-infected subjects naive to ART presented with oral-mucosal lesions [22]. In Nigeria, Taïwo et al. reported that before 2005, 36.4 % of HIV-infected women in Nigeria presented with a clinical diagnosis of oral candidiasis [23]. However, the prevalence of oral candidiasis in our study was higher than the one reported by Owotade et al. in South Africa where 12.8 % of HIV- infected women on ART were diagnosed with oral candidiasis [24]. The relatively high prevalence of oral-mucosal lesions (23.8 %) in our HIV-infected population accessing ART raised questions concerning ART efficacy or adherence that need to be explored.

The prevalence of carious lesions was elevated in our sample, although not associated with HIV infection. The DMFT index was moderate, with a high prevalence of carious lesions. In addition, few FSW were classified as having a good oral hygiene. The DMFT index reported in our study was higher than the DFMT index reported (2.7) in the study conducted in 2004 by Samba et al. in the general population of Abidjan from 18 to 59 years [25]. This study included all socio-professional strata among whom junior and senior executives had a more accurate perception of their own health and recognized the need to look after their oral health. Indeed, the number of dental consultations increased with the level of education [26, 27]. The high prevalence of periodontitis found in HIV-infected FSW was in accordance with a report from N'diaye et al. conducted in Senegal among FSW. In their study, the prevalence of periodontitis was 26.1 % in HIV- infected FSW not on ART and 2.5 % in HIV-negative FSW [28].

The presence of periodontitis in our study was significantly associated with HIV infection as already reported in industrialized countries [29, 30]. In 2013, Pattrapornnan P. et al. reported an association between the occurrence of periodontitis and a decline in CD4 counts [31]. In our study, nearly half of HIV-positive FSW had CD4 count <350 cell/mm^3^, which might partly explain the high prevalence of periodontitis. However, mechanisms leading to periodontal disease in HIV-infected people were multiple [32–34]. Another known factor associated with periodontitis is tobacco use [35]. The absence of association, in our study, between tobacco use and periodontitis might be related to the relatively short median duration of smoking. Indeed, the impact of tobacco smoking on the periodontal tissue is dose-dependent and needs time to induce periodontitis [36–38]. Tobacco and alcohol consumption were particularly high in our study. The prevalence of tobacco smoking reported here was higher than the estimated 1.1 % prevalence reported in the general female population in Côte d’Ivoire in 2002 [39]. Our results were also higher than those reported by Jaquet et al. among HIV-positive women on ART in west Africa [13]. This difference might be linked to socio-behavioural factors specific to sex work, which are frequently associated with the use of addictive product [40]. Therefore, it is important to perform a longitudinal follow-up of this population that might develop these diseases later. In addition, awareness and prevention measures should be directed towards excessive alcohol and tobacco use. Stress was another factor associated with the presence of periodontitis in previous reports. Stigma and discrimination associated with HIV/AIDS are widespread in Africa. FSW live in an extremely difficult climate of stigma related to sex work profession and HIV status as well as human rights violations [41]. Stress related to stigma might partly explain the association between periodontitis and HIV infection.

Our study has several limitations. The clinical diagnosis of oral diseases, especially oral-mucosal lesions might be operator-dependent. Prior to the study, a training on the diagnosis of oral manifestations of HIV infection was followed by the dental surgeon who performed the clinical oral examination. This training module was designed and developed by the Oral HIV/AIDS Research Alliance (http://www.aidsresearch.org/). There may be a potential biases in the assessment of oral diseases. In particular, periodontitis was assessed through a specific method developed by the WHO. The existence of alternative methods might limit comparisons with other studies. The study sample might not be representative of FSW in Abidjan. Indeed, FSW already in care for their HIV infection might be more likely to attend the clinic. Nevertheless, there were no particular criteria for FSW to attend the clinic. In addition, the prevalence of HIV infection reported in our randomly selected sample is quite similar to previous findings among FSW in Abidjan and elsewhere in sub-Saharan Africa. The “Clinique de Confiance” is the largest centre for health promotion, prevention and care targeting FSW in Côte d’Ivoire. For logistical reasons, we were also unable to document several important factors such as exposure to refined carbohydrates or HIV viral load in HIV-infected FSW which might have played a major role in the occurrence of oral lesions. Finally, the cross-sectional nature of our study did not allow us to draw any causal relation between oral diseases and their associated factors.

## Conclusion

This study showed a high prevalence of oral diseases among FSW in Abidjan. HIV infection was frequent and significantly associated with the presence of periodontal disease and oral-mucosal lesions. Facing the importance of stigma among FSW in sub-Saharan Africa, there is therefore a need to conduct additional studies to assess the impact of stigma on the occurrence of periodontitis in this population.

As oral diseases have a negative impact on the quality of life and can adversely affect various vital functions, there is a need to deliver prevention and care services for oral diseases in most vulnerable population such as FSW in sub-Saharan Africa.

## Additional file

Additional file 1:
**Appendix 1.** (PDF 135 kb)

## Abbreviations

ART: Antiretroviral therapy
AUDIT: Alcohol use disorders identification test
CDC: Centers for Disease Control
CI: Confidence Interval
CPI: Community Periodontal Index
DMFT: Decayed, missing and filled teeth
FSW: Female sex worker
HIV: Human Immunodeficiency Virus
IeDEA: International Epidemiologic Databases to Evaluate AIDS
IQR: Interquartile Range
OHIS: Oral Hygiene Index Simplified
OR: Odds ratio
SD: Standard deviation
WHO: World Health Organization
