# Supplementary material for: Oral health and HIV infection among female sex workers in Abidjan, Côte d’Ivoire
Source: BMC Oral Health. 2015 Dec 2;15:154. doi: 10.1186/s12903-015-0129-0 (PMC4668627; doi:10.1186/s12903-015-0129-0)
Supplement: Additional file 1: — Appendix 1. (PDF 135 kb) [file 12903_2015_129_MOESM1_ESM.pdf]

Appendix 1

The IeDEA West Africa Collaboration Study Group (as of July 28, 2015):
Participating sites (*members of the **Steering Committee,** §members of the **Executive Committee**):

Benin, Cotonou:
Adults: Djimon Marcel Zannou*, Carin Ahouada, Jocelyn Akakpo, Christelle Ahomadegbe, Jules Bashi, Alice Gougounon-Houeto, Angele Azon-Kouanou, Fabien Houngbe, Jean Sehonou (CNHU Hubert Maga).
Pediatrics: Sikiratou Koumakpai*§, Florence Alihonou, Marcelline d'Almeida, Irvine Hodonou, Ghislaine Hounhoui, Gracien Sagbo, Leila Tossa-Bagnan, Herman Adjide (CNHU Hubert Maga).

Burkina Faso:
Adults: Joseph Drabo*, Rene Bognounou, Arnaud Diendere, Eliezer Traore, Lassane Zoungrana, Beatrice Zerbo (CHU Yalgado, ***Ouagadougou***), Adrien Bruno Sawadogo*§, Jacques Zoungrana, Arsene Hema, Ibrahim Sore, Guillaume Bado, Achille Tapsoba (CHU Souro Sanou, ***Bobo Dioulasso***)

Pediatrics: Diarra Ye*, Fla Koueta, Sylvie Ouedraogo, Rasmata Ouedraogo, William Hiembo, Mady Gansonre (CH Charles de Gaulle, ***Ouagadougou***).

Côte d'Ivoire, Abidjan:
Adults: Eugene Messou*, Joachim Charles Gnokoro, Mamadou Kone, Guillaume Martial Kouakou, (ACONDA-CePReF); Clarisse Amani Bosse*, Kouakou Brou, Achi Isidore Assi (ACONDA-MTCT-Plus); Henri Chenal*, Denise Hawerlander, Franck Soppi (CIRBA); Albert Minga*, Yao Abo, Jean-Michel Yoboue (CMSDS/CNTS); Serge Paul Eholie*§, Mensah Deborah Noelly Amego, Viviane Andavi, Zelica Diallo, Frederic Ello, Aristophane Koffi Tanon (SMIT, CHU de Treichville), Serge Olivier Koule*, Koffi Charles Anzan, Calixte Guehi (USAC, CHU de Treichville);

Pediatrics: Edmond Addi Aka*, Koffi Ladji Issouf, Jean-Claude Kouakou, Marie-Sylvie N'Gbeche, (ACONDA-CePReF); Toure Pety*, Divine Avit-Edi (ACONDA-MTCT-Plus); Kouadio Kouakou*, Magloire Moh, Valerie Andoble Yao (CIRBA); Madeleine Amorissani Folquet*, Marie-Evelyne Dainguy, Cyrille Kouakou, Veronique Tanoh Mea-Assande, Gladys Oka-Berete, Nathalie Zobo, Patrick Acquah, Marie-Berthe Kokora (CHU Cocody); Tanoh Francois Eboua*, Marguerite Timite-Konan, Lucrece Diecket Ahoussou, Julie Kebe Assouan, Mabea Flora Sami, Clemence Kouadio (CHU Yopougon).

Ghana, Accra:
Pediatrics: Lorna Renner*§, Bamenla Goka, Jennifer Welbeck, Adziri Sackey, Seth Ntiri Owiafe (Korle Bu TH).

Guinea-Bissau:
Adults: Christian Wejse*§, Zacarias Jose Da Silva*, Joao Paulo (Bandim Health Project), The Bissau HIV cohort study group: Amabelia Rodrigues (Bandim Health Project), David da Silva (National HIV program Bissau), Candida Medina (Hospital National Simao Mendes, Bissau), Ines Oliviera-Souto (Bandim Health Project), Lars Ostergaard (Dept of Infectious Diseases, Aarhus University Hospital), Alex Laursen (Dept of Infectious Diseases, Aarhus University Hospital), Morten Sodemann (Dept of Infectious Diseases, Odense University Hospital), Peter Aaby (Bandim Health Project), Anders Fomsgaard (Dept. of Virology, Statens Serum Institut, Copenhagen), Christian Erikstrup (Dept. of Clinical Immunology), Jesper Eugen-Olsen (Dept. of Infectious Diseases, Hvidovre Hospital, Copenhagen).

Mali, Bamako:
Adults: Moussa Y Maiga*§, Fatoumata Fofana Diakite, Abdoulaye Kalle, Drissa Katile (CH Gabriel Toure), Hamar Alassane Traore*, Daouda Minta*, Tidiani Cisse, Mamadou Dembele, Mohammed Doumbia, Mahamadou Fomba, Assetou Soukho Kaya, Abdoulaye M Traore, Hamady Traore, Amadou Abathina Toure (CH Point G).

Pediatrics: Fatoumata Dicko*, Mariam Sylla, Alima Berthe, Hadizatou Coulibaly Traore, Anta Koita, Niaboula Kone, Clementine N'Diaye, Safiatou Toure Coulibaly, Mamadou Traore, Naichata Traore (CH Gabriel Toure).

Nigeria:
Adults: Man Charurat* (UMB/IHV), Samuel Ajayi*, Georgina Alim, Stephen Dapiap, Otu (UATH, ***Abuja***), Festus Igbinoba (National Hospital ***Abuja***), Okwara Benson*, Clement Adebamowo*, Jesse James, Obaseki, Philip Osakede (UBTH, ***Benin City***), John Olasode (OATH, ***Ile-Ife***).

Senegal, Dakar:
Adults: Moussa Seydi*, Papa Salif Sow, Bernard Diop, Noel Magloire Manga, Judicael Malick Tine§, Coumba Cisse Bassabi (SMIT, CHU Fann),
Pediatrics: Haby Signate Sy*, Abou Ba, Aida Diagne, Helene Dior, Malick Faye, Ramatoulaye Diagne Gueye, Aminata Diack Mbaye (CH Albert Royer).

Togo, Lomé:
Adults: Akessiwe Patassi*, Awerou Kotosso, Benjamin Goilibe Kariyare, Gafarou Gbadamassi, Agbo Komi, Kankoe Edem Mensah-Zukong, Pinuwe Pakpame (CHU Tokoin/ Sylvanus Olympio).
Pediatrics: Annette Koko Lawson-Evi*§, Yawo Atakouma, Elom Takassi, Ameyo Djeha, Ayoko Ephoevigah, Sherifa El-Hadj Djibril (CHU Tokoin/Sylvanus Olympio).

Executive Committee*: Francois Dabis (Principal Investigator, Bordeaux, France), Emmanuel Bissagnene (Co-Principal Investigator, Abidjan, Cote d'Ivoire), Elise Arrive (Bordeaux, France), Patrick Coffie (Abidjan, Cote d'Ivoire), Didier Ekouevi (Abidjan, Cote d'Ivoire), Antoine Jaquet (Bordeaux, France), Valeriane Leroy (Bordeaux, France), Charlotte Lewden (Bordeaux, France), Annie J. Sasco (Bordeaux, France).

Operational and Statistical Team: Jean-Claude Azani (Abidjan, Cote d'Ivoire), Eric Balestre (Bordeaux, France), Serge Bessekon (Abidjan, Cote d'Ivoire), Camille Gilbert (Bordeaux, France), Sophie Karcher (Bordeaux, France), Jules Mahan Gonsan (Abidjan, Cote d'Ivoire), Jerome Le Carrou (Bordeaux, France), Severin Lenaud (Abidjan, Cote d'Ivoire), Celestin Nchot (Abidjan, Cote d'Ivoire), Karen Malateste (Bordeaux, France), Amon Roseamonde Yao (Abidjan, Cote d'Ivoire), Bertine Siloue (Abidjan, Cote d'Ivoire).

Administrative Team: Gwenaelle Clouet (Bordeaux, France), Madikona Dosso (Abidjan, Cote d'Ivoire), Alexandra Doring§ (Bordeaux, France), Adrienne Kouakou (Abidjan, Cote d'Ivoire), Elodie Rabourdin (Bordeaux, France), Jean Rivenc (Pessac, France).
